# Behavioral Health Student Training and Intervention in the Community-Based Mobile Health and Wellness Program

**DOI:** 10.1093/geroni/igaf122.653

**Published:** 2025-12-31

**Authors:** Rachel Regal, Leland Waters, Patricia Slattum

**Affiliations:** Virginia Commonwealth University, Richmond, Virginia, United States; Virginia Commonwealth University, Richmond, Virginia, United States; Virginia Commonwealth University College of Health Professions, Richmond, Virginia, United States

## Abstract

The Mobile Health and Wellness Program (MHWP) trains interprofessional students on 4Ms age-friendly care as part of care coordination and health promotion weekly clinics for medically underserved older adults within low-income senior housing buildings and a community-based wellness clinic. Of the nearly 3000 visits in 2024 with 374 older adults, student teams from nursing, medicine, pharmacy, and other disciplines provided behavioral health intervention in 436 visits (15%) with over a third of participants (n = 131). Unmet mental health needs identified in 56% (41 of 73) of screens for depression, anxiety, substance use, and social connection led to further assessment, mental health education, wellness goal setting, and care referrals or coordination. Interprofessional students engage with behavioral health training as part of team debriefings, morning topic discussions, a simulation orientation with motivational interviewing skill development, morning topic discussions on a range of mental health topics, and online 4Ms evidence-based training modules including trauma-informed care. Modules conclude with knowledge check questions and self-rated confidence on three key domains: identifying risk factors, conducting screenings, and intervention. Descriptive results indicate that the majority of students report confidence gains following each module. Results of Wilcoxon signed rank analyses showed a 1-rank median confidence increase for Mentation, Social Connectedness, and Ageism Modules (p < .001, N = 132 to 544). Further findings and details will be presented that describe how MHWP trains students and intervenes for behavioral health needs in community-based settings with underserved older adults.

